# Autoimmune pancreatitis mimicking carcinoma of the head of the pancreas: a case report

**DOI:** 10.1186/1752-1947-6-11

**Published:** 2012-01-11

**Authors:** Mohammad-Reza Ghadir, Farshad Sheikhesmaili, Fatemeh Attari, Reza Safdari, Amirhossein Ghanooni, Maryam Vaez-javadi

**Affiliations:** 1 Department of Internal Medicine, Qom University of Medical Sciences, Shahid Beheshti General Hospital, Qom, Iran; 2 Digestive Diseases Research Center, Sanandaj University of Medical Sciences, Sanandaj, Iran; 3 Digestive Diseases Research Center, Tehran University of Medical Sciences, Tehran, Iran; 4 151 8th Alley, 18th Alley, Fatemi Street, Qom, Iran; 5 372 2nd Alley, Aqaqia Street, Qom, Iran; 6 Department of Immunology, Qom University of Medical Sciences, Qom, Iran

## Abstract

**Introduction:**

We report on a case of autoimmune pancreatitis presenting as pancreatic head cancer, which is extremely rare in Iran. Currently, on the PubMed database, no such cases exist.

**Case presentation:**

A 70-year-old Iranian man presented with recurrent abdominal pain, jaundice and elevated bilirubin and alkaline phosphatase levels. An abdominal computed tomography scan revealed a heterogeneous presence in the pancreatic head as well as dilated intra- and extrahepatic bile ducts. A common bile duct stent had been inserted. Our patient was subsequently diagnosed with pancreatic head cancer.

Due to his continued recurrent abdominal pain, our patient returned to the hospital. His levels of bilirubin, alkaline phosphatase and tumor markers were all normal but his immunoglobulin G4 and antinuclear antibodies were extremely high. A biopsy of the pancreatic head heterogeneity by endoscopic ultrasonography was performed.

Pathologic samples showed fibrosis associated with lymphoplasmacytic infiltration and no evidence of malignancy. A diagnosis of autoimmune pancreatitis was confirmed, the bile duct stent removed, and an appropriate treatment plan was undertaken.

**Conclusion:**

Autoimmune pancreatitis should be considered in suspected cases of pancreatic cancer. In these instances, a biopsy of the pancreas will help to differentiate between the two and prevent complications due to disease progression as well as unnecessary surgery.

## Introduction

Autoimmune pancreatitis (AIP) is a rare disease and has only been reported in certain countries [1]. Cases of autoimmune pancreatitis that mimic pancreatic head cancer are rare in Iran.

AIP is a primary pancreatic disorder that is associated with other autoimmune disorders, such as primary biliary cirrhosis, rheumatoid arthritis, sarcoidosis, inflammatory bowel disease and vitiligo [2]. The disease commonly presents as recurrent mild abdominal pain with obstruction of the biliary ducts and pancreatic duct; there may be a pancreatic mass that can be misdiagnosed as pancreatic lymphoma or cancer [3]. Although such cases are rare, an accurate diagnosis of AIP can prevent the progression of the disease and unnecessary surgery.

The diagnostic criteria defined by the Mayo Clinic include one or more of three characteristics: diagnostic histology; related features in abdominal magnetic resonance imaging (MRI) or computed tomography (CT) in association with increased levels of immunoglobulin G4 (IgG4); and the response of intra- or extrapancreatic manifestations to corticosteroid therapy [4].

The imaging features of the disease have similar presentations in both MRI and CT scans and may include local or diffuse enlargement of the pancreas [5]. AIP can appear as a common bile duct stricture in an endoscopic retrograde cholangiopancreatography. It is impossible to distinguish AIP from pancreatic cancer solely by means of such imaging techniques [6].

A serum level of IgG4 above 135 ng/dL has very high sensitivity and specificity in differentiating AIP from pancreatic cancer [7]. However, although increased serum levels of IgG4 and radiologic features associated with a therapeutic response to corticosteroids are suggestive of AIP, a pancreatic biopsy is essential in confirming the diagnosis. Histological findings in the pancreatic sample include dense fibrosis and inflammatory levels of lymphoplasmacytic infiltration [4].

## Case presentation

**Figure 1:**
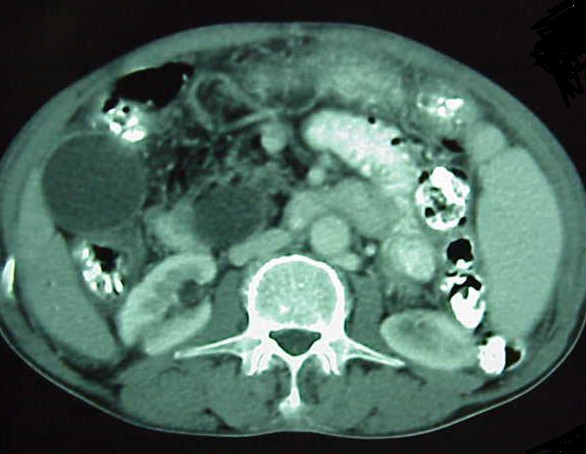
**CT scan before treatment**. Dilatation of extra- and intrahepatic bile ducts and heterogeneity of the head of the pancreas are visible.

**Figure 2:**
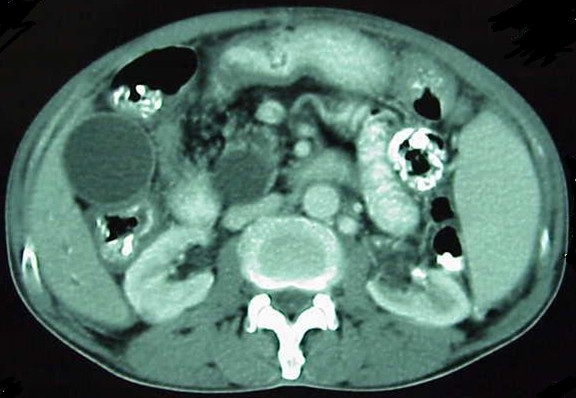
**CT scan before the treatment**. See the heterogeneity of the head of pancreas.

Our patient was a 75-year-old Iranian man, admitted to hospital with recurrent upper abdominal pain for the past 18 months. A common bile duct plastic stent had been inserted based on the results of diagnostic investigations, including an obstructive pattern of liver enzyme elevation, dilatation of extra- and intrahepatic bile ducts revealed through ultrasonography and heterogeneity of the pancreatic head (likely due to cancer) in an abdominal spiral CT scan with oral- and venous-contrast media (Figures 1 and 2). No abnormalities were found during a physical examination, with the exception of mild upper abdominal tenderness and vitiligo patches on his neck and hands (Tables 1 and 2).

**Table 1:** The results of laboratory tests before the stent placement.

Product	Level
Aspartate aminotransferase	110 U/L
Alanine aminotransferase	120 U/L
Alkaline phosphatase	540 IU
Total bilirubin	4.5 mg/dL
Direct bilirubin	2.5 mg/dL
Amylase	450 IU

**Table 2:** The laboratory test results after the stent placement.

Test	Level
Aspartate aminotransferase	21 U/L
Alanine aminotransferase	10 U/L
Alkaline phosphatase	250 IU
Total bilirubin	0.6 mg/dl
Direct bilirubin	0.2 mg/dl
Amylase	100 IU
White blood cell count	4100 cells/mm
Hemoglobin	311 g/dL
Mean corpuscular volume	76 fL
Erythrocyte sedimentation rate	36 mm/h
Blood urea nitrogen	18 mg/dL
Creatinine	1 mg/dL
Serum iron	25 mg/dL
Total iron binding capacity	468
Immunoglobulin G4	187 ng/mL
Carcinoembryonic antigen	2.7 ng/mL
Carbohydrate antigen 125	16 ng/mL
Carbohydrate antigen 19-9	8 ng/mL
Alpha fetoprotein	2.5 ng/5 mL

**Figure 3:**
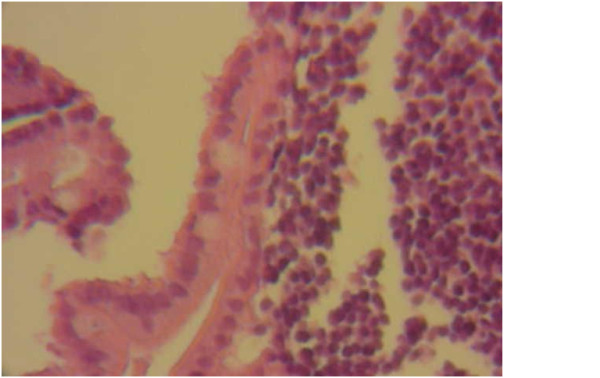
**Pathologic evaluation of the pancreas biopsy specimen**. The specimen shows fibrosis and lymphoplasmacytic infiltration without evidence of malignancy.

**Figure 4:**
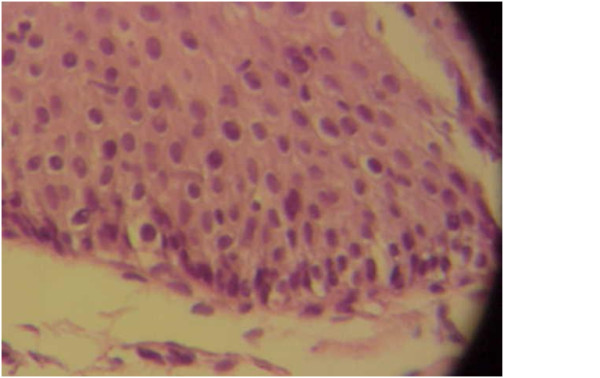
**Pathologic findings of the pancreas biopsy specimen**. Lymphoplasmacytic infiltration of the sample is visible.

An upper gastrointestinal endoscopy, aimed at controlling the presence of occult blood in his stool, iron deficiency anemia and heartburn, showed lower esophageal ulcers associated with diaphragmatic herniation. A pathologic evaluation of the ulcer biopsy specimens confirmed reflux esophagitis. A colonoscopy was normal. Mild dilatation of his extra- and intrahepatic bile ducts was seen in repeated abdominal ultrasonography procedures. However, an endoscopic ultrasound showed a hypoechoic area, 2 cm in size, in the head of his pancreas. The pathological and cytological results of an aspiration biopsy of the lesion revealed fibrosis and inflammatory cell infiltration without evidence of malignancy (Figures 3 and 4).

Once AIP had been diagnosed, prednisolone was administered. Two months after treatment, a reevaluation of the pancreas head by means of an abdominal spiral CT scan with oral and venous contrast media did not show any abnormality, and the common bile duct stent was removed because of the positive therapeutic response.

## Discussion

The etiology of AIP, a rare type of chronic pancreatitis, has been recognized by clinical, histological and morphologic findings [3]. Recently, it has been described as a systemic disease with lymphoplasmacytic infiltration that is associated with other autoimmune diseases. The disease has been reported in a number of countries and its incidence has been rising in some regions, especially in Japan [1,6].

Typically, AIP can present in various clinical forms, including mild pancreatitis, acute recurrent pancreatitis, biliary duct strictures and pancreatitis, with such clinical presentations as primary sclerosing cholangitis and a pancreatic mass, which may be misdiagnosed as pancreatic cancer.

Acute AIP symptoms may also include rare, intermittent abdominal pain, weight loss, jaundice and an obstructive pattern of liver enzymes (an inappropriate rise of alkaline phosphatase relative to a mild elevation in aminotransferases) that may also be seen in cases of pancreatic cancer and cholangiocarcinoma [8].

AIP has been reported in two patients with obstructive jaundice and a diagnosis of pancreatic head cancer was made based on radiologic studies conducted by Japanese researchers [9]. In another study, AIP was reported as the most common benign disease in patients with a diagnosis of pancreatic adenocarcinoma who had undergone a pancreatoduodenectomy [10].

The definitive diagnosis of AIP is made by radiologic CT scan findings of a narrowing of the pancreatic duct and parenchymal edema of the pancreas [8]; and on MRI as a focal or diffuse expansion of the pancreas (a sausage shape). These findings are usually associated with an absence of vascular marking, calcification or fluid collection around the pancreas, a narrowing of the main pancreatic duct and a crescent development of the pancreas head [5].

AIP is routinely responsive to corticosteroid therapy over a one to four month period [7]. During treatment, patients typically have an immunological follow-up with an assessment of serum levels of IgG4 through a CT scan.

## Conclusion

AIP is an autoimmune disease with varying diagnostic criteria, which include a range of pancreatic manifestations and specific MRI findings. A pancreatic biopsy, increased IgG4 serum level and a therapeutic response to corticosteroids are necessary in determining a definitive differentiation from a diagnosis of pancreatic adenocarcinoma based on radiologic findings [4]. A precise diagnosis of AIP would prevent complications arising from the progression of the disease and unnecessary surgery [4,10].

## Consent

Written consent was obtained from the patient for publication of this case report and any accompanying images.

## Competing interests

The authors declare that they have no competing interests.

## Authors' contributions

GM conducted the first approach and examinations, participated in the treatment of our patient and oversaw the manuscript to publication. He was also responsible for final approval and supervision of the manuscript. SF and AF were involved in taking our patient's history and revising the manuscript. SR contributed to the writing and translation of the manuscript. GA contributed to the editing of the final manuscript. All authors read and approved the final manuscript.
